# Case Reports: Trial Dysphagia Interventions Conducted via Telehealth

**DOI:** 10.5195/ijt.2016.6193

**Published:** 2016-12-15

**Authors:** STACY GALLESE CASSEL

**Affiliations:** DEPARTMENT OF COMMUNICATION DISORDERS, STOCKTON UNIVERSITY, GALLOWAY, NJ, USA

**Keywords:** Oropharyngeal dysphagia, Tele-dysphagia, Telehealth

## Abstract

The diagnosis of dysphagia, defined as swallowing dysfunction or difficulty, is estimated to affect 40–60% of the institutionalized geriatric population, and is the leading cause of aspiration pneumonia, one of the primary contributors of geriatric mortality. In the United States, statistics suggest that at least 50% of these individuals have limited access to treatment due to mobility, distance, and socioeconomic constraints. While “tele-dysphagia intervention” – the delivery of dysphagia therapy services via telecommunications technology – may provide a solution, there is limited research investigating its validity or reliability. The following three case reports of individuals successfully participating in trial tele-dysphagia therapy sessions lend credibility to this service delivery approach, and highlight the need for future research.

Therapeutic intervention for dysphagia, defined as swallowing dysfunction or difficulty (Logemann, 1998), is primarily provided by speech-language pathologists to maintain adequate nutrition and respiratory safety, as well as the quality of life associated with oral intake (Gustafsson & Tibbling,1991; Logemann, 1998).

It is suggested in the literature that swallowing disorders are effectively managed via intensive dysphagia therapy that incorporates motor learning (Shigematsu, Fujishima, and Onoh, 2013; Lan et al 2012; Crary et al, 2010; Schmidt & Lee, 1988) as well as principles of activity dependent neuroplasticity (e.g., therapy that targets intensity, saliency, and specificity) (Kleim & Jones, 2008).

Subsequently, speech-language pathologists specializing in dysphagia anticipate an increasing role in diagnosis and intervention (Coyle, 2012) given the growing geriatric population and likelihood of an increase in the incidence of dysphagia. In rural and socioeconomically challenged areas, however, access to such intervention remains limited due to distance, mobility challenges, and the unavailability of qualified speech-language pathologists to provide dysphagia services (Coyle, 2012). The term “tele-dysphagia” has emerged as a result of recent efforts to employ and research the merging of telepractice and dysphagia (Coyle, 2012). Although there is a growing base of evidence validating the use of tele-dysphagia for assessment purposes (Malandraki, et al., 2011, 2013; Ward, Burns, Theodoros, & Russell, 2014), minimal research exists to validate the use of tele-dysphagia for therapeutic intervention (Ward & Burns, 2014), with two exceptions: a single pediatric case study featuring an intensive feeding program (Malandraki, Roth, & Sheppard, 2014), and a study of adults diagnosed with head and neck cancer (although the focus on the latter was on multi-disciplinary team collaboration rather than direct intervention) (Burns, et al., 2012). Given the potential benefit of tele-dysphagia therapeutic intervention, the purpose of this investigation was to determine whether formal dysphagia goals could be met using online tele-dysphagia.

## CASE REPORTS

### PARTICIPANTS

Three patients with a formal medical diagnosis of dysphagia each participated in an individual trial intervention session with the investigating speech-language pathologist. Dysphagia may occur in either the oral, pharyngeal, or esophageal stages of the swallow, defined below:

*ORAL STAGE* – During this stage, the food is chewed, broken down by saliva, and formed into a bolus which is propelled to the back of the back of the mouth/oral cavity. (University of Wisconsin Hospitals and Clinics Authority, 2016).
*PHARYNGEAL STAGE* – During this stage, the food is propelled through the pharynx toward the esophagus/ stomach via muscular contraction, which allows for protection of the airway by the closure of the vocal folds and the movement of the epiglottis over the airway. Pathology at this stage could result in aspiration pneumonia (University of Wisconsin Hospitals and Clinics Authority, 2016).
*ESOPHAGEAL STAGE* – During this stage, the bolus enters the esophagus from the pharynx and is propelled into the stomach by muscular contraction (University of Wisconsin Hospitals and Clinics Authority, 2016).

The case history and clinical presentation of each participant follows:

### PARTICIPANT 1

A 59-year old male had a primary medical diagnosis of an acute left hemisphere cerebral vascular accident (CVA). A clinical dysphagia assessment, followed by a videofluoroscopic swallowing assessment, revealed the following:

#### ORAL STAGE –

Reduced, prolonged oral transit time was observed (with all tested consistencies (ground and pureed solids, honey, nectar and thin liquids) secondary to right oro-facial hypotonia, as characterized by a right ocular droop and right extra/intra-oral weakness, Given ten trials, the oral transit time ranged from 3.5 to 5.5 seconds. Diffuse residue of both puree and solid ground consistencies was noted throughout the oral cavity following the onset of the swallow response, predominantly in the right buccal space secondary to right-sided oral weakness consistent with a left CVA. Research indicates that such oral residue presents an increased risk of aspiration (Molfenter and Steele, 2013). The participant was able to clear the bolus from the oral cavity when instructed to use a liquid wash for 100% of all opportunities. Additional oral findings were unremarkable.

#### PHARYNGEAL STAGE --

A pharyngeal swallow delay was observed for all trials (range: 2 – 3 seconds) with premature posterior leakage of all tested consistencies to the level of the vallecular space secondary to decreased tongue base retraction and reduced bilateral pharyngeal contraction. There was no evidence of penetration or aspiration; vallecular retention of ground and pureed solids was observed for approximately 30–50% of each bolus trial. Research indicates that pharyngeal residue presents an increased risk of aspiration (Molfenter and Steele, 2013). It was noted that the liquid wash reduced the vallecular retention by ≥50% for all ground and solid trials.

#### ESOPHAGEAL STAGE –

Findings were unremarkable at the time of assessment.

### PARTICIPANT 2

A 62-year old male had a primary diagnosis of acute traumatic brain injury (TBI) and subsequent dysphagia. A clinical dysphagia assessment, followed by a videofluoroscopic swallowing assessment, revealed the following:

#### ORAL STAGE –

Reduced, prolonged oral transit time was observed secondary to bilateral oro-facial hypotonia with all tested consistencies (pureed solids, honey, nectar and thin liquids); given 14 trials, oral transit time ranged from 2.5 to 4.5 seconds; diffuse oral residue was noted for 6 of 14 trials and was cleared with an independently initiated second swallow.

#### PHARYNGEAL STAGE --

Excessive pharyngeal vallecular residue (bolus of food lodged in the valleculae of the pharynx) was noted with all tested consistencies (increased with pureed solids) secondary to decreased tongue base retraction and reduced bilateral pharyngeal contraction. No aspiration or penetration was observed, however, research indicates that vallecular residue presents an increased risk of aspiration (Molfenter & Steele, 2013). It was observed that the subsequent use of a chin down postural compensation (refer below – TREATMENT) reduced the vallecular residue for 4 of 5 trials conducted during the assessment.

#### ESOPHAGEAL STAGE --

Findings were unremarkable at the time of assessment.

### PARTICIPANT 3

A 74-year old female had a primary medical diagnosis of acute left CVA and subsequent dysphagia. A clinical dysphagia assessment, followed by a videofluoroscopic swallowing assessment, revealed the following compromise:

#### ORAL STAGE --

Reduced, prolonged oral transit time was observed with all tested consistencies (ground and pureed solids, honey, nectar and thin liquids) secondary to oro-facial hypotonia (right>left); given 16 trials, oral transit time ranged from 2.0 to 3.5 seconds; Diffuse oral residue was noted for 5 of 16 trials and was cleared with independently initiated subsequent swallow(s).

#### PHARYNGEAL STAGE –

Excessive pharyngeal residue was noted with all tested consistencies in the pyriform sinuses bilaterally (increased on the right side), superior to the cricopharyngeal junction; this was secondary to a delayed triggering of the swallow response and bilaterally reduced pharyngeal contraction. Residue was reduced in 5 of 6 trials following the use of a right head turn postural compensation. Penetration was observed during the swallow secondary to reduced airway closure for all thin liquid trials with an absent sensory response; no aspiration was observed. It should be noted that pharyngeal residue presents an increased risk of aspiration (Molfenter & Steele, 2013).

#### ESOPHAGEAL STAGE --

Mildly delayed emptying of the esophagus was noted with all tested consistencies (range= 14–17 seconds).

### INTERVENTIONS

The objective of the investigation was to determine whether the goals typically targeted in the in-person setting were achieved when addressed via tele-dysphagia. The individual goals of all three trial sessions focused on training the patient to independently use “swallowing safety strategies.” Such strategies, as defined by ASHA, incorporate “modifications in food/liquid bolus size, bolus texture, patient positioning, compensatory maneuvers, and sensory enhancement techniques” to facilitate optimal safety and efficiency during oral intake, as well as to minimize aspiration risk (ASHA, 2002). Such strategies are employed by speech-language pathologists and taught to patients following a clinical dysphagia assessment, the results of which are used to determine the clinical strategy.

It should be noted that although the use of postural compensations can be used to address swallowing safety at the time of assessment and intervention, such strategies have not been determined to provide long-term safety (Johnson, Herring, & Daniels, 2014) post-discharge.

For Participant 1, the CYCLIC INGESTION strategy was recommended to promote swallowing safety given the presence of oral residuals and vallecular residue. The strategy involves alternating liquids and solids during oral intake at a specified ratio (e.g., ingesting one teaspoon of a solid followed by one teaspoon of liquid) to promote adequate bolus clearance from the oral, pharyngeal, and esophageal areas, thus minimizing aspiration risk. As noted above, research indicates that pharyngeal residue presents an increased risk of aspiration, and the effectiveness of cyclic ingestion was determined in the course of assessment.

For Participant 2, the CHIN DOWN positional strategy was recommended to promote swallowing safety. This strategy involves touching the chin to the neck prior to initiation of the swallow, which allows more time for the vocal folds to close, reducing the risk of aspiration (Welsh, et al, 1993). The chin down position also been suggested as a compensatory strategy to reduce vallecular residue secondary to reduced BOT retraction to the posterior pharyngeal wall (Welsh, et al, 1993). As noted above, research indicates that pharyngeal residue presents an increased risk of aspiration, and the effectiveness of the chin down posture was determined in the course of assessment.

For Participant 3, the HEAD ROTATION positional strategy was recommended to promote swallowing safety. The HEAD ROTATION strategy (which involves turning the head horizontally to either the right or left) rotates the pharynx so that the bolus flows to the stronger side of the pharynx in the cases of pharyngeal hemiparesis for safer bolus transfer (Johnson, Herring, & Daniels, 2014). This posture additionally pushes the opposing side toward the midline, therefore improving adduction of the vocal folds. Lastly, it opens the cricopharyngeal muscle to reduce pharyngeal residuals that would otherwise invade the airway (Johnson, Herring, & Daniels, 2014). As noted above, research indicates that pharyngeal residue presents an increased risk of aspiration, and the effectiveness of the head turn compensation was determined in the course of assessment.

Each trial tele-dysphagia session was conducted between the clinician and the participant (targeting the strategies outlined above) using the Macintosh FaceTime videoconferencing system to allow for a real-time interaction, as well as VSee, a HIPAA-compliant, encrypted telehealth application that ensured participant confidentiality (VSee, 2015). The participants were given a brief orientation to the equipment and needed to demonstrate sufficient ability (as determined by clinical judgment) to hear the audio signal and respond to commands. All sessions were recorded and analyzed with written consent of the participants. Each session took place during a mealtime, with the clinician cueing (via visual or auditory cues) the participant in the use of the recommended swallowing safety strategies. Data was collected for the first 30 trials of each session, during which the participant self-presented each bolus. Participant responses for each trial were judged to be positive or negative (positive meaning the participant was able to successfully employ the strategy and negative meaning the participant was NOT able to employ the strategy).

So as to determine the effectiveness of the tele-dysphagia session, each strategy was addressed in a formal therapy goal consistent with an in-person session, with the outcome measure being achievement of the goal in the tele-dysphagia session. The goals for each participant were as follows:

*PARTICIPANT 1* – The patient will perform the cyclic ingestion strategy during each self-presented bolus trial, given gradually fading visual-auditory cues, with 80% accuracy.
*PARTICIPANT 2* – The patient will perform the chin down strategy during each self-presented bolus trial, given gradually fading visual-auditory cues, with 80% accuracy.
*PARTICIPANT 3* – The patient will perform the right head rotation strategy during each self-presented bolus trial, given gradually fading visual-auditory cues, with 80% accuracy.

The tele-dysphagia session was the third session for each patient; the first two sessions were conducted in the in-person mode (also recorded and analyzed with written consent of the participant). Three sessions were conducted to compare the in-person and tele-dysphagia methods.

## RESULTS

Findings revealed that, in the course of 3 sessions (the first two in the in-person mode and the third via tele-dysphagia), all participants effectively achieved their formal therapy goals in both settings. Goal achievement in the first two in-person sessions (session 1 providing baseline data) is reported in Table 1

To determine inter-rater reliability, a second speech-language pathologist viewed all of the sessions and also judged whether participant responses were positive or negative. Additionally, when comparing in-person findings with tele-dysphagia findings, the participants were able to maintain or surpass their goals in the tele-dysphagia session, providing preliminary evidence suggesting the effectiveness of tele-dysphagia for intervention.

Inter-rater reliability scores were obtained via the calculation of a Pearson correlation coefficient, to measure the degree of agreement between the two raters for each trial over three sessions with three participants. Findings indicated that the correlation coefficient was 1, which indicates perfect level of agreement between the two raters. Goal achievement during tele-dysphagia session is reported in Table 2

## DISCUSSION

The term “tele-dysphagia” has emerged as a result of recent efforts to employ and research the merging of telepractice and dysphagia (Coyle, 2012), and use of swallowing safety strategies for the prevention of aspiration has been well-documented in numerous studies (Johnson, Herring, & Daniels, 2014; Welsh, et al, 1993; Macrae, Anderson, & Humbert, 2014; Fraser & Steele, 2012) -- although additional evidence is needed to establish the long-term benefits of both. To the knowledge of this investigator, no tele-dysphagia studies have been conducted on the validity of therapeutic intervention with cognitively compromised adults. The findings of this study may provide some preliminary data indicating the efficaciousness of tele-dysphagia as a method of treatment, or to substantiate the need for additional studies; for example, a larger sample size with random assignment into in-person and tele-dysphagia groups.

## CONCLUSION

The findings of these case studies suggest the potential for tele-dysphagia to be used as a successful delivery method for therapeutic intervention, given that all three participants successfully achieved their goals and were able to maintain or surpass their level of achievement (when compared to results from the in-person session) during the tele-dysphagia session. These findings were confirmed by inter-rater reliability scores. It should be noted that the strategies targeted in the participant goals are consistently utilized by clinicians (Johnson, Herring, & Daniels, 2014). Again, establishing the long-term benefits (e.g., the ongoing prevention of aspiration, and thus, respiratory safety) of the instruction and use of swallowing strategies via tele-dysphagia intervention has yet to be determined, and warrants further research. These findings are indeed preliminary, since only three cases were reported, which limits the ability to generalize findings to other populations or disorders. Additionally, analysis was performed on only the first 30 trials, the minimum number completed by all participants. Further research should target a larger sample size and a broader range of populations, increased trials, long-term benefits, and most importantly, an ongoing comparison of in-person and tele-dysphagia interventions. The results of this investigation provide preliminary evidence that can justify such future studies, and represent initial steps in efficacious use of tele-dysphagia for intervention.

## Figures and Tables

**Table 1:** Goal Achievement during In-person Dysphagia Sessions

Participant	In-person goal	In-person Session 1 Goal achievement accuracy	In-person Session 2 Goal achievement accuracy
1	The patient will demonstrate use of the cyclic ingestion strategy during each self-presented bolus trial, given gradually fading visual-auditory cues, with 80% accuracy.	80% of 30 trials (24 total) with cues utilized 90% of all trials (27 total)	80% of 30 trials (24 total) with cues utilized for 90% of all trials (27 total)
2	The patient will demonstrate use of the chin down strategy during each self-presented bolus trial, given gradually fading visual-auditory cues, with 80% accuracy.	70% of 30 trials (21 total) with cues utilized for 100% of all trials (30 total)	86.67% of 30 trials (26 total) with cues utilized for 90% of all trials (27 total)
3	The patient will demonstrate use of the right head rotation strategy during each self-presented bolus trial, given gradually fading visual-auditory cues, with 80% accuracy.	60% of 30 trials (18 total) with cues utilized for 100% of all trials (30 total)	80% of 30 trials (24 total) with cues utilized for 90% of all trials (27 total)

**Table 2:** Goal Achievement during Tele-dysphagia Session

Participant	Trial tele-dysphagia goal (Session 3)	Accuracy achieved
1	The patient will demonstrate use of the cyclic ingestion strategy, given gradually fading visual-auditory cues, with 80% accuracy.	90% of 30 trials (27 total) with cues utilized 70% of all trials (21 total)
2	The patient will demonstrate use of the chin down strategy, given gradually fading visual-auditory cues, with 80% accuracy.	86.67% of 30 trials (26 total) with cues used 100% of all trials (30 total)
3	The patient will demonstrate use of the head turn strategy, given gradually fading visual-auditory cues, with 80% accuracy.	83.33% of 30 trials (25 total) with cues utilized 90% of all trials (27 total)
